# Therapeutic Potential of *Polyscias fruticosa* (L.) Harms Leaf Extract for Parkinson's Disease Treatment by *Drosophila melanogaster* Model

**DOI:** 10.1155/2022/5262677

**Published:** 2022-05-19

**Authors:** Hai Trieu Ly, Thi Thu Huong Nguyen, Van Minh Le, Bich Thao Lam, Thi Thu Trinh Mai, Thi Phuong Thao Dang

**Affiliations:** ^1^Research Center of Ginseng and Medicinal Materials (CGMM), National Institute of Medicinal Materials, Ho Chi Minh City 700000, Vietnam; ^2^Faculty of Biology and Biotechnology, University of Science, Vietnam National University-Ho Chi Minh City, Ho Chi Minh City 700000, Vietnam; ^3^Vietnam National University-Ho Chi Minh City, Vietnam

## Abstract

Parkinson's disease (PD) is characterized by progressive locomotive defects and loss of dopaminergic neurons. *Polyscias fruticosa* leaves are used by Vietnamese as herbal medicines to support the treatment of some diseases related to neurodegeneration such as Parkinson's and Alzheimer's diseases. However, recent scientific data have not provided sufficient evidence for the use of *P. fruticosa* leaves to treat PD or decelerate PD progression. In the present study, the capacity of *P. fruticosa* leaf extract for PD treatment on the dietary supplementation was investigated using dUCH-knockdown *Drosophila* model. The results indicated that *P. fruticosa* leaf extract decelerated dopaminergic neuron degeneration induced by dUCH knockdown in not only the larval stage but also the adult stage, which might result in the amelioration in locomotor ability of dUCH-knockdown larvae and flies. Furthermore, antioxidant activities and some key phytochemicals such as saponins, polyphenols, and flavonoids that might contribute to the effects of the *P. fruticosa* leaf extract were identified.

## 1. Introduction

*Polyscias fruticosa* (L.) Harms is a plant species included in the Araliaceae family and is used as a medicinal plant with great therapeutic potential worldwide. *P. fruticosa* possesses some pharmacological effects similar to some *Panax* species such as enhancing the body's immunity system, improving male fertility, preventing fatigue, nourishment, increasing appetite, sleeping well, increasing work capacity, gaining weight, and antidote [1, 2]. This plant can be a substitution for its costlier counterpart in curing many diseases. In Vietnamese folk medicine, *Polyscias fruticosa* leaves were also used to assist in the treatment of neurodegenerative diseases such as Parkinson's and Alzheimer's diseases because of improving symptoms of tremor, loss of balance, insomnia, memory impairment, nervous tension, and nervous breakdown. Pharmacological studies have shown that *P. fruticosa* had other extensive effects such as antidepressant, antistress, improve memory, antioxidant, hypoglycemic, hepatoprotective, hypolipidemic, antifungal, and antibacterial effects [2–9].

*P. fruticosa* has been reported to contain saponins, alkaloids, glycosides, polyphenols, flavonoids, tannins, vitamins (C, B_1_, B_2_, and B_6_), and amino acids [2, 3]. Previous studies showed that saponins have been considered the main constituents of *P. fruticosa*; however, a number of other components, such as flavonoids and polyphenols, are also found in this species [3], which are very typical secondary metabolizing compounds with antioxidant effects in plants. Previous publications also showed the *in vitro* and *in vivo* antioxidant activities of *P. fruticosa* leaves [4, 6, 7].

Parkinson's disease (PD) is one of the most common neurodegenerative diseases particularized by the loss of dopaminergic neurons. As a result, a decrease in the dopamine (DA) neurotransmitter in the striatum leads to descent in mobility [10]. PD affects around 1-3% of the population aged over 65, and the number of patients with PD will accrete from 8.7 to 9.3 million by 2030 [11]. The PD pathology is related to some motor defects and several nonmotor symptoms [12]. Although the etiology of PD is still largely unknown, previous studies suggested that the cause involves multifactorial factors such as genetics, environmental agents, and aging [13, 14]. Among them, factors that lead to oxidative stress may play a role in the development and progression of PD. Thus, phytochemical constituents and antioxidant properties of *P. fruticosa* leaves may be therapeutic for PD.

Currently, there is no effective treatment since therapeutic drugs that do not prevent disease progression, for example, levodopa or dopaminergic agonists, have been only used to relieve motor symptoms by restoring neurotransmitters. However, the long-term use of these medications can lead to serious side effects such as fatigue symptoms and other motor complications [13, 15]. Thus, it is vital that scientists develop new neuroprotective agents that are intended to decrease or preclude the progression of PD. It is difficult to access the human brain to validate a new agent's ability to treat. Therefore, scientists have researched and developed a variety of experimental models using animals and in vitro culture cells that could mimic different aspects of PD. One of the animal models that can simulate PD symptoms to not only study the mechanism of PD but also to screen for therapeutic drugs is the *Drosophila* model [16]. One among *Drosophila* model for PD, a PD fly model by knocking down dUCH (*Drosophila* ubiquitin carboxyl-terminal hydrolase), a homologous gene of UCH-L1 in humans, in DA neurons was established [17]. This model could display pathophysiological features and mimic PD symptoms including mobility defects such as difficulty in walking, slow movement, tremor, and progressive DA neuron degeneration [12, 17]. Furthermore, knockdown of dUCH resulted in increased levels of ROS leading to oxidative stress and antioxidant agents consisting of vitamin C, curcumin, and *Portulaca oleracea* extract were demonstrated to improve the PD-like phenotypes in this fly model [18–20]. Therefore, this model might be useful for screening agents with therapeutic effects on Parkinson's disease.

Hence, based on the pharmacological actions and chemical composition of *P. fruticosa* leaves, we hypothesized that *P. fruticosa* leaves might improve motor capacity and neurodegeneration in PD. To elucidate this issue, the current study aimed to determine the effects of *P. fruticosa* leaf extract in PD treatment using the *Drosophila* model of PD induced by knocking down dUCH. We found that *P. fruticosa* leaf extract had the potential to protect against the dopaminergic neuron degeneration induced by dUCH knockdown in not only the larval stage but also the adult stage. This might result in improvements in PD-like phenotypes which are characterized by progressive locomotive defects of dUCH-knockdown larvae and flies.

## 2. Materials and Methods

### 2.1. Plant-Based Material and Extract Preparation

The leaves *Polyscias fruticosa* (L.) Harms were collected in GACP-WHO (Good Agricultural and Collection Practices-World Health Organization) certified growing area in May 2019 from Nghia Hung District, Nam Dinh Province, Vietnam. Leaves were randomized on mature plants about 3 years old and without any distinction between young or old leaves. The leaves were picked and processed according to GACP-WHO standards by TRAPHACO Joint Stock Company, Vietnam. These dried leaves were ground to a fine powder and kept individually in an airtight PVE bag at the Research Center Ginseng and Medicinal Materials in Ho Chi Minh City (Sample code: PFL-TTS-2019).

The dried leaf powder was extracted with 45% ethanol in the ratio of 1:15 (weight: volume) at room temperature for 24 hours in the percolator apparatus. The liquid extract was collected at a rate of 2 mL/min and concentrated using a rotary evaporator at 60°C under reduced pressure to obtain *P. fruticosa* leaf extract (PLE). The extraction efficiency achieved was 27.25%. The crude extract was stored at 2-8°C for further experiments and limited exposure to light, heat, and air during the extraction process and all experiments.

#### 2.1.1. *Drosophila* Stocks

Wild-type strain Canton-S was obtained from the Bloomington Drosophila Stock Center (BDSC) was used to create control flies. RNAi line carrying *UAS-dUCH-IR* fusion (GD#26468) for knocking down *Drosophila ubiquitin carboxyl-terminal hydrolase* (*dUCH*, CG4265) was received from the Vienna Drosophila Resource Center (VDRC). dsRNAs produced from the RNAi line are specific for *dUCH* in *Drosophila melanogaster*. *TH-GAL4* (tyrosine hydroxylase) driver (BDSC#8848) (*ple-GAL4*) was used to specifically knock down *dUCH* in dopaminergic neurons. Control flies were generated by crossing Canton-S with the *TH-GAL4* driver.

#### 2.1.2. *Drosophila* Culture and Crosses

Fly stocks were cultured on standard food containing 5% dry yeast, 5% sucrose, 3% powdered milk, and 0.85% agar at 25°C. *P. fruticosa* extract and vitamin C (L-Ascorbic acid, Sigma-Aldrich, CAS 50-81-7, ≥98% purity by HPLC, positive control) were dissolved in distilled water and then added to standard food at suitable concentrations. They were maintained in the dark to protect against the loss of antioxidant activity during experimental procedures. *P. fruticosa* extract was added to the diet at final concentrations of 1, 2, 4, 8, and 16 mg/mL. The PD flies were also exposed to 5 mM of vitamin C. The control flies were separately allowed feeding on the selected doses of *P. fruticosa* extract and vitamin C. In this study, all tests using the GAL4/UAS system were effectuated at 28°C [20].

### 2.2. Analysis of *P. fruticosa* Leaf Extract through UV-Vis and HPLC

#### 2.2.1. Determination of Total Polyphenol Content

The total polyphenol content was estimated by Folin-Ciocalteu's method in which using gallic acid as a standard [21]. To briefly illustrate, 200 *μ*L test sample was mixed with 500 *μ*L of Folin-Ciocalteu's reagent (Merck) in 6 mL of double-distilled water. Then 1.5 mL of sodium carbonate (Merck) solution (20% w/v) was poured into this mixture right after 5 min, and with distilled water, the volume reached up to 10 mL. The reaction was kept in less-exposure-to-lights condition for 2 hours at room temperature. The absorbance was measured at 758 nm, and all determinations were made in triplicate. The calibration curve was planned to use standard gallic acid (Sigma-Aldrich, CAS 149-91-7, ≥97% purity by HPLC). Through the calibration plot, the total polyphenols content was calculated and expressed as milligram of gallic acid equivalent (GAE) per 100 milligrams of dry mass.

#### 2.2.2. Determination of Total Flavonoid Content

The total flavonoid content was determined based on the aluminum chloride colorimetric method in which quercetin (Sigma-Aldrich, CAS 117-39-5, ≥95% purity by HPLC) was used as a reference compound [21]. Dissolving 1.0 mg quercetin in 10 mL methanol (Merck) by which the stock solution was made, then by serial dilutions, including using methanol, the standard quercetin solutions were prepared. Briefly, 1 mL of aluminum chloride (Merck) (2% w/v) was added to 1 mL diluted extract or standard quercetin solutions separately, and with methanol, the mixture was made up to 10 mL in quantity. Then, the solution was mixed and incubated for 15 min at room temperature. The absorbance of the mixtures was calibrated at 416 nm with a UV-Vis spectrometer. The measurements were carried out in triplicate. The calibration curve was plotted using standard quercetin. The total flavonoids content was estimated from the calibration plot and expressed as milligram of quercetin equivalent (QE) per gram of dry mass.

#### 2.2.3. Quantification of Total Saponins in *P. fruticosa* Extract Using Oleanolic Acid Standard

The total saponin content was estimated by HPLC method with some minor adjustments in which using oleanolic acid (Sigma-Aldrich, CAS 508-02-1, ≥97% purity by HPLC) as a standard [22]. Oleanolic acid was dissolved in methanol (Merck) at 1 mg/mL to form a stock solution. The stock solution was continuously diluted to produce different concentration ranges (100-400 *μ*g/mL), which were filtered through a 0.45-*μ*m membrane filter before HPLC analysis.

One gram of crude extract of *P. fruticosa* leaves was dissolved in 100 mL methanol containing 2% HCl and extracted by reflux extraction for 8 h. The collected extract was concentrated under reduced pressure to get dry extract which nextly extracted with chloroform solvent by liquid-liquid extraction. The chloroform extracts were evaporated to gain dry chloroform extract and then adjusted to a volume of 5 mL by methanol and continuously filtered through a 0.45-*μ*m membrane filter before being injected into the HPLC system. The analysis was repeated 3 times to obtain the average mean.

Venusil XBP C18 column (4.6 × 250 mm, 5 *μ*m) with a C18 guard column was performed with acetonitrile (Merck) and water (the rate of 1: 9 v/v) in a gradient mode at a flow rate of 1.0 mL/min at 215 nm, and the separation was performed at 24°C.

The total saponin content was calculated and expressed as milligram of oleanolic acid equivalent per gram of dry mass through the regression equation was calculated in the form of *y* = *ax* + *b*, where *y* and *x* were peak area and compound concentration.

### 2.3. Antioxidant Activity Assays

#### 2.3.1. DPPH Radical Scavenging Assay

The DPPH free radical quenching assay was applied to evaluate the antioxidant activity of *P. fruticosa* leaf extracts based on a previously described method. Briefly, 2 mL reaction mixture consisting of 0.25 mL of different concentrations of test extract or positive control and 0.25 mL of 0.6 mM DPPH reagent (Sigma-Aldrich, CAS 1898-66-4, ≥95% purity by HPLC) in methanol was incubated at room temperature for 30 min in the dark. The absorbances were measured at 516 nm using a Spectro UV-2550 spectrophotometer. Ascorbic acid (vitamin C, Sigma-Aldrich, ≥98% purity by HPLC) was used as a positive control [21]. The control for each sample and negative control were also done.

In order to evaluate the antioxidant accumulation in larvae, larval extract of each strain was prepared by rubbing 50 larvae with 500 *μ*L of distilled water, and the mixtures were then centrifuged at 4°C for 20 minutes to collect the supernatant. The larval extract solutions were diluted at 1/2, 1/4, 1/8, and 1/16 serial dilutions to react with DPPH. The reaction took place in the dark for 30 min at room temperature. The absorbances were also recorded at 516 nm [20].

The percent DPPH inhibition was calculated by the equation:

(1) $$\begin{array}{c} Inhibition\;\left(\%\right)=\frac{A_{0}-\left(A_{1}-A_{2}\right)}{A_{0}}\times 100, \end{array}$$

where *A*_0_, *A*_1_, and *A*_2_ are the absorbance of the negative control (with DPPH solution and without test extract), the sample (with DPPH solution and test extract), and the control sample (without DPPH solution and with test extract), respectively.

#### 2.3.2. ABTS Radical Cation Decolorization Assay

The ABTS antioxidant test was carried out according to the following description. To start, a 7 mM ABTS (Sigma-Aldrich, CAS 30931-67-0, ≥98% purity by HPLC) solution was added to a 2.45 mM potassium persulfate solution, and the solution was incubated in the dark for 16 hours at room temperature. Then, the solution was diluted by mixing 1 mL of ABTS solution with 50 mL of methanol to obtain an absorbance of 0.70 ± 0.05 units at 734 nm using a spectrophotometer (Spectro UV-2550). Next, 20 *μ*L of the test sample at various concentrations or positive control was mixed with 980 *μ*L of ABTS solution, and the absorbance was measured at 734 nm after 6 min at room temperature. All samples were done in triplicate, and an average of each sample was calculated. Ascorbic acid was used as a positive control [21]. The percent ABTS inhibition was calculated by the equation:

(2) $$\begin{array}{c} Inhibition\;\left(\%\right)=\frac{A_{0}-\left(A_{1}-A_{2}\right)}{A_{0}}\times 100, \end{array}$$

where *A*_0_, *A*_1_, and *A*_2_ are the absorbance of the blank (with ABTS solution and without test extract), the sample (with ABTS solution and test extract), and the control sample (without ABTS solution and with test extract), respectively.

#### 2.3.3. Reducing Power Assay

The reducing property of the extract was determined by assessing the ability of the extracts to reduce FeCl_3_ solution as described by Oyaizu [23]. The mixture including 0.2 mL aliquot with 0.5 mL of sodium phosphate buffer 200 mM (pH 6.6) and 0.5 mL potassium ferricyanide 1% was incubated at 50°C for 30 min, then added 0.5 mL10% trichloroacetic acid (Sigma-Aldrich, CAS 76-03-9, ≥99% purity by HPLC), and centrifuged at 3000 rpm for 10 min. The volume of 0.5 mL of the supernatant was mixed with an equal volume of double-distilled water and 0.1 mL ferric chloride 0.1%. The absorbance was measured at 700 nm. Ascorbic acid was used as a positive control. The FRAP was subsequently analyzed via optical density and EC_50_ values. The lower the optical density value, the weaker the reduction activity of the sample. The EC_50_ value is the concentration of effective antioxidants for the absorbance reaches 0.5, which was calculated through the equation illustrating the correlation between the concentration of the sample and its optical density.

#### 2.3.4. Lipid Peroxidation Assay

Thiobarbituric acid reactive species (TBARS) assay was used to measure the lipid peroxide formed, using mouse brain homogenates as lipid-rich media. Malondialdehyde (MDA), a secondary product of the oxidation of polyunsaturated fatty acids, reacts with two molecules of thiobarbituric acid (TBA), yielding a pinkish-red chromogen with an absorbance maximum at 532 nm [24]. Brain homogenate (500 *μ*L, in 5 mM phosphate buffer) and 100 *μ*L of extract were mixed in a test tube, and the volume was made up to 2 mL, and by adding phosphate buffer, the mixture was incubated for 15 min at 37°C. Thereafter, 1 mL 10% TCA was added to stop the reaction, and then the mixture was centrifuged at 10000 rpm for 5 min. The mixture including 2 mL organic upper layer was mixed with 1 mL 0.8% TBA (Sigma-Aldrich, CAS 504-17-6, ≥98% purity by HPLC) and was heated in a boiling water bath for 15 min. The absorbance of the mixture was measured at 532 nm. For the blank, 100 *μ*L of distilled water was used in place of the extract. Trolox (Calbiochem Ltd., Co., CAS 53188-07-1, Purity ≥98% by titration) was used as a positive control.

The percent lipid peroxidation inhibition was calculated by the equation:

(3) $$\begin{array}{c} Inhibition\;\left(\%\right)=\frac{A_{0}-\left(A_{1}-A_{2}\right)}{A_{0}}\times 100, \end{array}$$

where *A*_0_, *A*_1_, and *A*_2_ are the absorbance of the blank (with TBA solution and without test extract), the sample (with TBA solution and test extract), and the control sample (without TBA solution and with test extract), respectively.

### 2.4. Growth and Survival Rates

Embryos of each fly strain were placed in a tube of the extract-containing medium at different concentrations including 0, 1, 2, 4, 8, and 16 mg/mL (each concentration was repeated three times). The number of pupae and flies formed was determined daily. The toxicity of the extract was assessed via the proportion of adult flies that were still alive after one day at each concentration compared to the untreated flies. The relative percentage of growth was calculated by the formula:

(4) $$\begin{array}{c} A=\frac{Number\;\text{of }adult\;flies\;e\text{x}tract\;treated}{Number\;\text{of }embryos\;picked\;extract\;treated}\times 100, \end{array}$$

(5) $$\begin{array}{c} B=\frac{Number\;\text{of }adult\;flies\;extract\;untreated}{Number\;\text{of }embryos\;picked\;extract\;untr\text{e}ated}\times 100, \end{array}$$

(6) $$\begin{array}{c} Growth\;relative\;\left(\%\right)=\frac{A}{B}\times 100. \end{array}$$

### 2.5. Feeding Assay

Feeding test was carried out to measure the amount of food that larvae have eaten over a period of time. The food intake was measured indirectly through the amount of a dye, Coomassie Brilliant Blue G-250 (#808274-10 g, Biomedicals, USA), and supplemented to medium consumed by larvae. The dye (2%, w/v) was added to the extract-containing standard medium. Early third-instar larvae at groups were collected and transferred into dye-containing food within 30 minutes. All larvae were then fed to tubes at a density of 10 larvae per tube, masticated samples in PBS, and centrifuged. The supernatant was collected, 10% ethanol was added, and absorbance was measured at 595 nm. The relative antioxidant intake was evaluated from the amount of extract-containing food that the larvae consumed at each concentration compared to the lowest concentration [20].

### 2.6. Crawling Assay

The motility of the larvae can be assessed through the crawling speed in order to be able to observe the effects of the extract on the early stage of the disease. Crawling assay was realized as described previously [25] with some slight alterations. Three male larvae in the third-instar stage were located at the center on a 2% agar Petri dish for each observation (*n* = 45). The movement of these larvae was filmed by a camera within 1 minute. These videos were analyzed using the ImageJ software (NIH, USA), and the wrMTrck plugin to get average speed data was then collected by Microsoft Excel 2016.

### 2.7. Negative Geotaxis Assay (Climbing Assay)

The climbing assay was performed as described previously [26]. Forty newly hatched adult male flies were taken into cylindrical tubes 20 cm high and 2 cm in diameter. The tubes were patted down five times to collect the flies in the bottom before recording the fly movements for one minute. These procedures were repeated five times and recorded by a digital camera. In this assay, the height to which each fly climbed after 5 seconds was scored as follows: 0 (less than 2 cm), 1 (between 2 and 4 cm), 2 (between 4 and 6 cm), 3 (between 6 and 8 cm), 4 (between 8 and 10 cm), 5 (between 10 and 12 cm), 6 (between 12 and 14 cm), 7 (between 14 and 16 cm), and 8 (more than 16 cm). The climbing index was collected from a repeated measurement and analyzed with a one-way ANOVA and graphed using GraphPad Prism 8.0.2.

### 2.8. Quantification of Dopaminergic Neurons by Immunostaining

Immunofluorescence was carried out following a standard protocol with several minor adaptations [27]. In this study, tyrosine hydroxylase (TH) enzyme was applied as a marker for DA neurons, which is a catalytic enzyme that converts L-tyrosine to a precursor of dopamine. Larval and adult brains were anatomized in cold phosphate-buffered saline (PBS) and fixed in 4% paraformaldehyde at 25°C for 20 minutes. The brains were blocked with 0.15% PBS-T (PBS containing 0.3% Triton X-100) containing 10% normal goat serum (blocking solution) at 25°C for 30 minutes after being washed with 0.3% PBS-T twice. All the brains were then incubated with a primary antibody, rabbit anti-TH (Millipore, AB152, Japan) and diluted in blocking solution (1: 250 v/v) at 4°C for 36 hours. Next, the brains were washed with 0.3% PBS-T and incubated with secondary antibodies conjugated with Alexa 488 (1: 500 v/v; Invitrogen) at 25°C for 2 hours. Brains were then washed and mounted in VECTASHIELD Mounting Medium (Vector Laboratories, Japan). Samples were taken by Nikon fluorescence microscopy (Nikon, Japan), and the number of DA neurons in each cluster was manually counted using the cell counter plugin of ImageJ 1.49o (NIH, USA).

### 2.9. Statistical Analysis

Data were collected by Microsoft Excel 2016 (Microsoft, USA). The average values were calculated, statistically analyzed, and graphed using GraphPad Prism 8.0.2 (Inc., La Jolla, CA, USA). Graphs were drawn using GraphPad Prism 8.0.2 with accordant algorithms. IC_50_ (inhibitory concentration, 50%) values were calculated from the mean values of data from three determinations based on the graph. The results were expressed in terms of mean ± SD (standard deviation), and data were analyzed by GraphPad Prism software using *t*-test and one-way ANOVA.

## 3. Results

### 3.1. Phytochemical Constituents and Antioxidant Activity of *P. fruticosa* Leaf Extract

Flavonoids have been widely reported to have an antioxidant effect [28]. The presence of polyphenol and flavonoid contents in *P. fruticosa* leaves suggested that *P. fruticosa* leaves extract (PLE) may have antioxidant capacity. Therefore, total polyphenol and flavonoid contents of PLE were then determined. The results showed that the total polyphenol and flavonoid contents of PLE in dry weight were 20.57 mg GAE/100 mg and 8.30 mg QE/g, respectively (Table 1).

**Figure 1:**
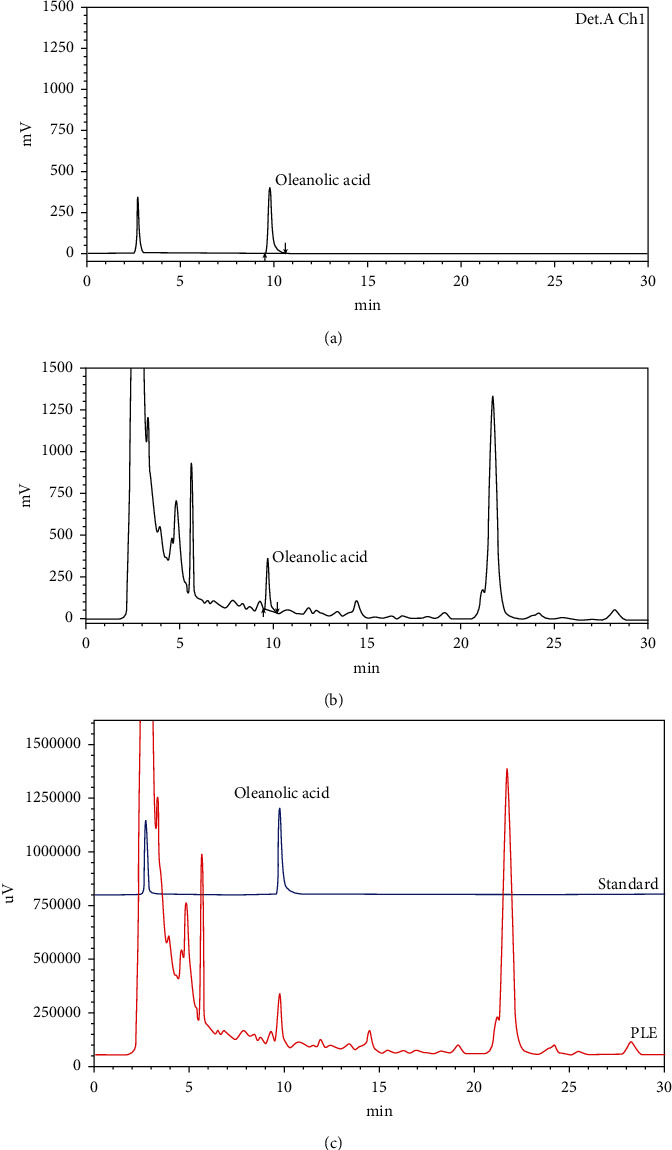
High-performance liquid chromatograms of standard and *P. fruticosa* leaf extract (PLE). The retention time of oleanolic acid reference standard (a), PLE (b), and their merge (c) peak were found to be at 9.7 min.

On the other hand, saponins are also one of the potential indicators for neuroprotective drug screening [29, 30]. Previous studies reported that saponins and saponin-containing herbs played a role in the prevention of dopaminergic neuron degeneration [31–33]. In this study, total saponin content of *P. fruticosa* leaves extract was determined by HPLC method using oleanolic acid as a standard. The results showed that total saponin content of *P. fruticosa* leaf extract was 23.5 mg/g dry weight (Table 1 and Figure 1).

Antioxidant activity of *P. fruticosa* leaf extract was evaluated in which concentration of PLE inhibited 50% of free radical; lipid peroxidation (IC_50_ value) or that reached 50% effective antioxidant activity (EC_50_ value) was determined. The results strongly demonstrated that *P. fruticosa* leaf extract has high antioxidant activity (Figure S1). The IC_50_/EC_50_ values of PLE were presented in Table 1 compared to the antioxidant capacity of vitamin C which was 3.61 ± 0.02 *μ*g/mL (DPPH inhibition), 9.06 ± 0.51 *μ*g/mL (ABTS inhibition), and 4.91 ± 0.12 *μ*g/mL (reducing power) and trolox which was 28.70 ± 0.95 *μ*g/mL (lipid peroxidation).

### 3.2. Toxicity of *P. fruticosa* Leaf Extract on *D. melanogaster*

**Figure 2:**
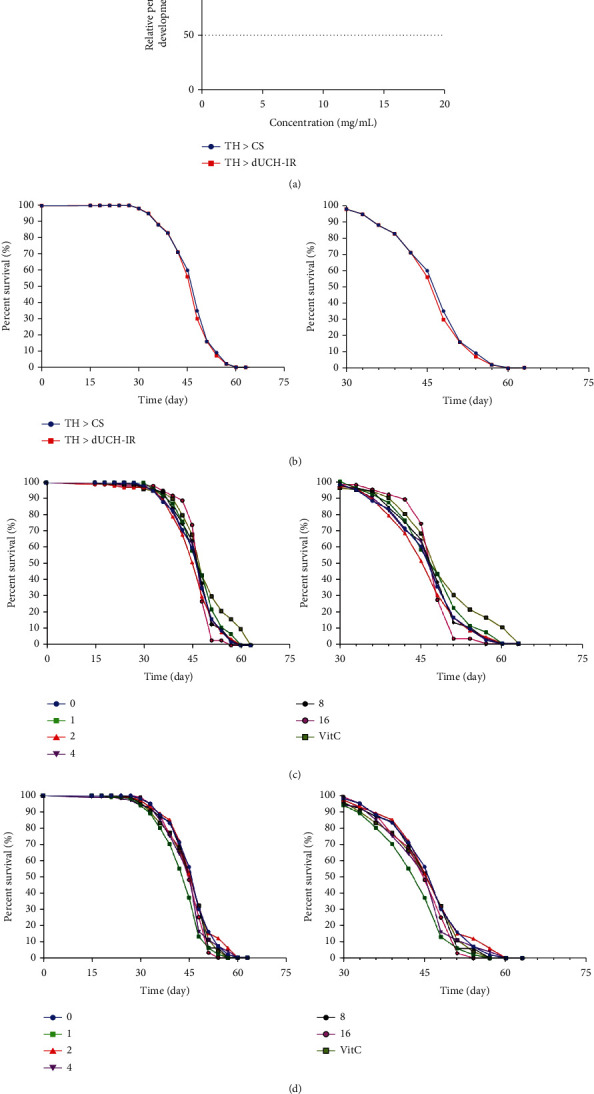
Effect of *P. fruticosa* leaf extract on survival rate of *D. melanogaster*. (a) Relative percent of development. Sample size *N* = 100 and biological replication *n* = 3; error bars represent the standard deviation of data. (b) Show survival rate of control flies compared to dUCH-knockdown flies for 63 consecutive days and from day 30 to 63, respectively (*n* =100); (c) show effect of PLE at different concentrations (0–16 mg/mL) on survival rate of control flies for 63 consecutive days and from day 30 to 63, respectively (*n* =100); (d) show effect of PLE at different concentrations (0–16 mg/mL) on survival rate of dUCH-knockdown flies for 63 consecutive days and from day 30 to 63, respectively (*n* =100); control line TH > CS (+; +; *TH-GAL4*/+) and dUCH-knockdown line TH > dUCH-IR (+; +; *TH-GAL4*/*UAS-dUCH-IR*). VitC: 0.5 mM vitamin C.

The toxicity of PLE on the development and viability of fruit flies was evaluated by analyzing the relative of growth and the relative of survival. The data showed that the relative growth of both control flies and dUCH-knockdown flies was not significantly changed when treated with PLE at 1, 8, and 16 mg/mL (Figure 2(a)).

The survival rate of flies from 1 day to 42 days of age was similar between dUCH-knockdown and control flies. By days 45 and 48, the survival percentage of flies in the control group decreased to 60% and 35% while 56% and 30% in the knockdown group, respectively (Figures 2(b)). This suggested that knockdown of dUCH slightly reduced *Drosophila* vitality. The results showed in Figures 2(c) and 2(d) also suggested that *P. fruticosa* leaf extract at treated concentrations did not have significant effects on survival rate compared to the untreated group.

### 3.3. Effects of *P. fruticosa* Leaf Extract on *D. melanogaster* Development

**Figure 3:**
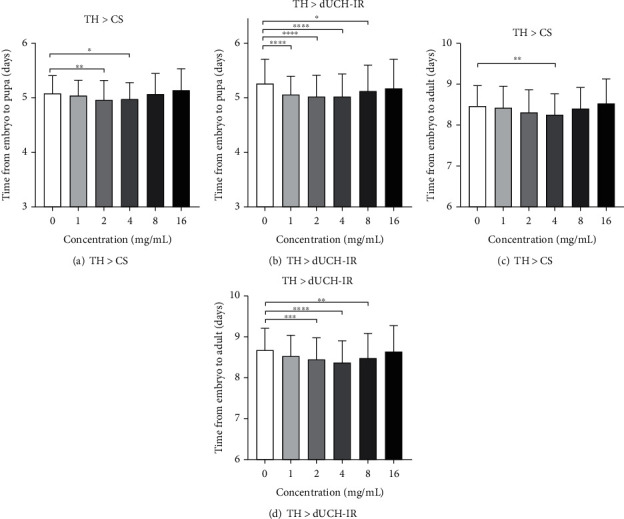
Effects of *P. fruticosa* leaf extract on *D. melanogaster* development. Control line TH > CS (+; +; *TH-GAL4*/+) and dUCH-knockdown line TH > dUCH-IR (+; +; *TH-GAL4*/*UAS-dUCH-IR*). (a, b) Period from embryo stage to pupa stage. (c, d) Period from embryo stage to adult stage. (*n*_*a*_ = 190, *n*_*b*_ = 198, *n*_c_ = 180, and *n*_*d*_ = 180; one-way ANOVA, Kruskal-Wallis test, ^∗^*p* < 0.05, ^∗∗^*p* < 0.01, ^∗∗∗^*p* < 0.001, and ^∗∗∗∗^*p* < 0.0001). Error bars represent the standard deviation of data.

To examine the effect of *P. fruticosa* leaf extract on fly development, time of each stage in fly life circle was assessed. In the stage from embryo to pupa, the PLE at concentrations of 2 and 4 mg/mL slightly reduced the fly growth time by 1.025 and 1.021 times, respectively, compared with that of untreated flies in the control fly group (Figure 3(a)). The PLE also slightly reduced growth time in the knockdown fly group at concentrations of 1–8 mg/mL (approximately 1.04 times) compared to untreated flies (Figure 3(b)). In the stage from embryo to adult fly, only the 4 mg/mL of the PLE reduced growth time of the flies 1.023 times compared to the untreated control in the control fly group (Figure 3(c)), while knockdown flies treated with 2–8 mg/mL of the PLE exhibited a decline in developmental time 1.024–1.036 times compared with untreated knockdown flies (Figure 3(d)).

### 3.4. Food and Antioxidant Intake Capacities of Larvae Treated with *P. fruticosa* Leaf Extract

**Figure 4:**
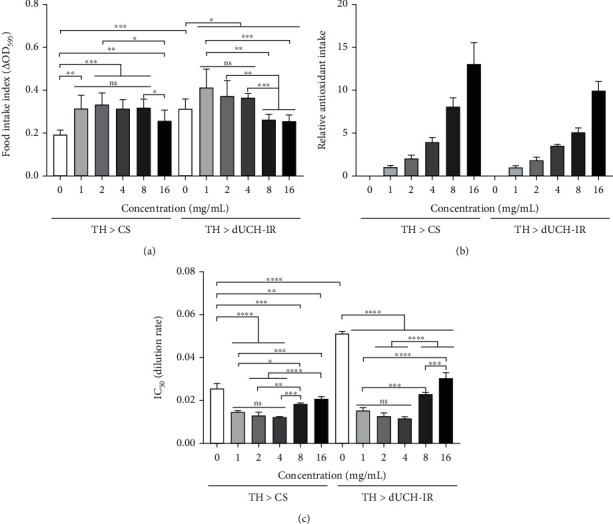
Food intake and antioxidant accumulating capacities of larvae when treated with *P. fruticosa* leaf extract. (a) Effect of PLE on the eating ability of third-instar larvae, *Δ*OD_595_: delta optical density at 595 nm. Population size *N* = 10 and biological replication *n* = 7; Mann-Whitney test, ^ns^*p* > 0.05, ^∗^*p* < 0.05, ^∗∗^*p* < 0.01, and ^∗∗∗^*p* < 0.001. (b) The relative antioxidant intake based on the amount of food consumed by larvae. Population size *N* = 10 and biological replication *n* = 7; Mann-Whitney test, ^∗∗∗^*p* < 0.001: significantly different pairs one by one in the same group. (c) The antioxidant accumulation by third-instar larvae via IC_50_ values of larval extract in DPPH assay. IC_50_ value was calculated by the dilution rate of larval extract at which the larval extract can convert 50% of DPPH radicals. Population size *N* = 50 and biological replication *n* = 3; *t*-test: ^ns^*p* > 0.05, ^∗^*p* < 0.05, ^∗∗^*p* < 0.01, ^∗∗∗^*p* < 0.001, and ^∗∗∗∗^*p* < 0.0001. The control line TH > CS (+; +; *TH-GAL4*/+) and the dUCH-knockdown line TH > dUCH-IR (+; +; *TH-GAL4*/*UAS-dUCH-IR*). Error bars represent the standard deviation of data.

Food and antioxidant intake of flies, when treated by PLE in serial concentrations from 0 to 16 mg/mL, were evaluated at the third-instar larvae stage. Results showed that the amounts of food which were ingested by treated knockdown and control larvae accreted remarkably when treated with PLE compared with untreated larvae. In the control larvae, there was no difference in larval feeding ability between groups treated with the PLE concentrations from 1 to 8 mg/mL. When larvae were treated with 16 mg/mL PLE, the food intake ability was significantly reduced compared to the concentration of 8 mg/mL. In the knockdown larvae, there was no difference in larval feeding capacity when treated with PLE at concentrations from 1 to 4 mg/mL. However, the ability decreased notably when larvae were treated with PLE at higher concentrations (8 and 16 mg/mL) compared with lower concentrations (Figure 4(a)).

The antioxidant intakes were enhanced by 1.81, 3.55, 5.06, and 9.95 fold when the knockdown flies were treated with 2, 4, 8, and 16 mg/mL PLE, respectively, compared to that of knockdown flies treated with 1 mg/mL PLE (Figure 4(b)).

In addition, after treatment with PLE, larval extract was also collected to analyze the antioxidant proficiency by DPPH assay to judge antioxidant accumulation of the larvae. Knockdown and control larvae treated with PLE at various concentrations were more likely to accumulate antioxidants (lower IC_50_ values) than the untreated larvae (Figure 4(c)). The antioxidant capacity of the larval extract increased gradually from a concentration of 0 to 4 mg/mL and decreased as the PLE concentration increased. This result was consistent with the results of food intake capacity of larvae obtained from Figure 4(a). The antioxidant ability of larval extracts when treated with PLE at 1, 2, and 4 mg/mL was significantly higher than that at the concentrations of 8 and 16 mg/mL.

### 3.5. *P. fruticosa* Leaf Extract Ameliorated Locomotor Dysfunction Caused by Knockdown of dUCH

There are many extracts and phytochemicals of medicinal herbs such as polyphenols, flavonoids, saponins, terpenoids, and alkaloids that have been shown to play roles in nerve protection, improving motor functions and cognitive performance, especially in Alzheimer's and Parkinson's diseases [29, 30]. Therefore, in this study *P. fruticosa* leaf extract was examined for its ability to improve motor function and reduce degeneration of dopaminergic neurons in the fly model.

**Figure 5:**
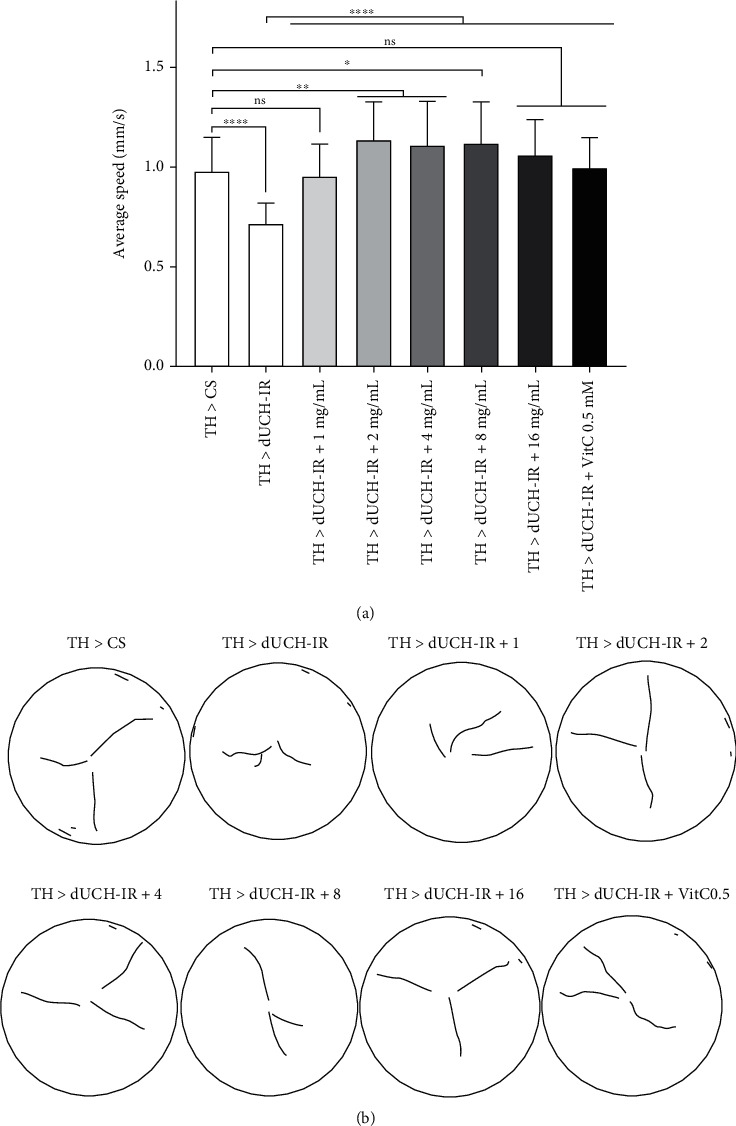
The effects of *P. fruticosa* leaf extract on the locomotive ability of larvae. (a) Average crawling speeds of third-instar larvae. The control line TH > CS (+; +; *TH-GAL4*/+) and the dUCH-knockdown line TH > dUCH-IR (+; +; *TH-GAL4*/*UAS-dUCH-IR*). Error bars represent the standard deviation of data. VitC: 0.5 mM vitamin C. *n* = 45, one-way ANOVA: Tukey's test: ^ns^*p* > 0.05, ^∗^*p* < 0.05, ^∗∗^*p* < 0.01, and ^∗∗∗∗^*p* < 0.0001. (b) Images show the motion paths of control larvae (TH > CS), dUCH-knockdown larvae (TH > dUCH-IR), and dUCH-knockdown larvae treated with the PLE at different concentrations (TH > dUCH-IR+ 1-16 mg/mL) and vitamin C (TH > dUCH-IR + VitC 0.5 mM).

The dUCH-knockdown flies have been demonstrated to have locomotor dysfunction in both larval and adult stages [17]. In this study, the effect of PLE on larval motility by crawling assay and adult flies by climbing assay was investigated. As a previous report [18], in this study, the dUCH-knockdown larvae exhibited not only abnormal behaviors but also decreased crawling speeds, and vitamin C at 0.5 mM could improve the motility of dUCH-knockdown larvae. Mobility of knockdown larvae (TH > dUCH-IR) increased after being treated with PLE at all treated concentrations. Specifically, the PLE at the concentrations of 2–8 mg/mL increased significantly the crawling speed of the dUCH-knockdown larvae compared to the control larvae (Figure 5(a)). Therefore, 2 mg/mL PLE was selected in further experiments.

Besides, dUCH-knockdown larvae crawled with tremor-like movement paths and the crawl paths were significantly shorter than that of the control larvae. The dUCH-knockdown larvae fed with the PLE displayed recoveries in crawling behavior (the crawl path is smoother and longer) (Figure 5(b)). As well as the effect of PLE on dUCH-knockdown larvae, the results of the effect of PLE on crawling ability and behavior of control larvae were also shown in Figure S2.

**Figure 6:**
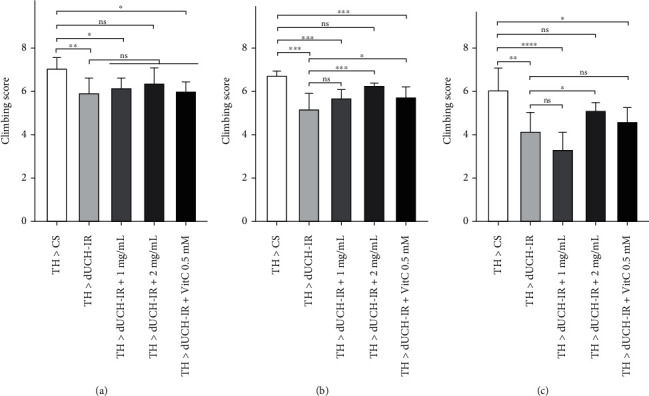
The effects of *P. fruticosa* leaf extract on the climbing ability of flies. The effects of long-term treatment with the PLE on climbing ability of 1-day-old (a), 5-day-old (b), and 10-day-old (c) adult flies. Long-term treatment: consecutively treating from embryos to 30-day-old flies. (a–c) Comparison of climbing score of control flies (TH > CS), dUCH-knockdown flies (TH > dUCH-IR), and dUCH-knockdown flies treated with the PLE at different concentrations (TH > dUCH-IR+ 1-2 mg/mL) and vitamin C (TH > dUCH-IR + VitC 0.5 mM) at surveyed times over 10 days. Population size *N* = 10 and biological replication *n* = 8; Mann-Whitney test, ns: not significant, ^ns^*p* > 0.05, ^∗^*p* < 0.05, ^∗∗^*p* < 0.01, ^∗∗∗^*p* < 0.001, and ^∗∗∗∗^*p* < 0.0001. The control line TH > CS (+; +; *TH-GAL4*/+) and the dUCH-knockdown line TH > dUCH-IR (+; +; *TH-GAL4*/*UAS-dUCH-IR*). Error bars represent the standard deviation of data. VitC: 0.5 mM vitamin C.

In adult fly, interestingly, amelioration of the locomotor dysfunction can also be observed when continuously treating the new hatched flies by PLE (Figure 6). At 1-day-old adult flies, the PLE and vitamin C 0.5 mM did not improve the climbing ability of the flies compared to the knockdown flies (Figure 6(a)). At 5-day-old adult flies, the PLE at the concentration of 2 mg/mL and vitamin C 0.5 mM ameliorated remarkably the treated-fly climbing capacity compared to the knockdown flies (Figure 6(b)). The PLE at this concentration also showed an improvement in treated-fly climbing ability compared to the knockdown flies at 10 days of age, while vitamin C 0.5 mM did not display the effect at this time (Figure 6(c)). Meanwhile, this effect of the PLE 1 mg/mL was not detected at all three-time points. Furthermore, the PLE at the examined concentrations and vitamin C at 0.5 mM had not shown effect on improving the climbing ability of dUCH-knockdown flies and did not affect the climbing ability of control flies at 5 days of age in the short treatment regimen (only larval stage) (Figure S3).

Taken together, the results suggested that *P. fruticosa* leaf extract had the effect of improving the mobility of flies expressing Parkinson's disease phenotype.

### 3.6. *P. fruticosa* Leaf Extract Decreased Degeneration of Dopaminergic Neurons Caused by Knockdown of dUCH

Impaired mobility is reported as a result of dopaminergic neuron (DA) damage or loss [34]. Previous research has shown that dUCH-specific DA knockdown caused randomly DA neuron degeneration in different clusters in the *Drosophila* model [17]. In this study, 2 mg/mL PLE could improve the crawling and climbing capabilities of dUCH-knockdown larvae and adult files, so it might play a role in protecting dopaminergic neurons. Hence, the effect of PLE on the degeneration of DA neurons in the dUCH-knockdown *Drosophila* was investigated at the concentration of 2 mg/mL through morphological analysis of DA clusters in the larvae and adult brains by immunostaining with anti-TH antibody, a marker of DA.

**Figure 7:**
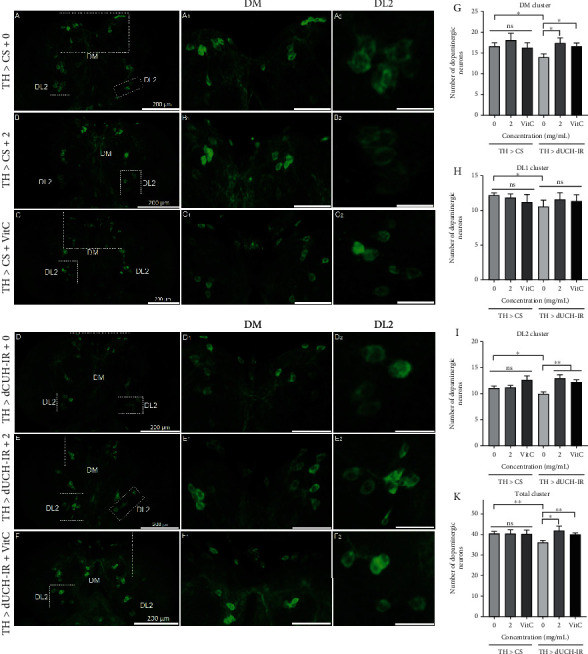
*P. fruticosa* leaf extract reduced degeneration of dopaminergic neurons caused by knockdown of dUCH in larvae. (a–f) Representative immunostaining images of brain lobes using antityrosine hydroxylase antibody. The control (TH > CS) and dUCH-knockdown larvae (TH > dUCH-IR) were treated with 2 mg/mL of PLE and 0.5 mM vitamin C. (A_1_-F_1_) and (A_2_-F_2_) show dopaminergic neurons in DM and DL2 clusters with scale bars of 50 and 25 *μ*m, respectively. The quantified data of the numbers of dopaminergic neurons in three clusters (DL1, DL2, and DM) and sum of three clusters was also presented in column charts (g-k). *n* = 8, *t*-test: ^ns^*p* > 0.05, ^∗^*p* < 0.05, and ^∗∗^*p* < 0.01. The control line TH > CS (+; +; *TH-GAL4*/+) and the dUCH-knockdown line TH > dUCH-IR (+; +; *TH-GAL4*/*UAS-dUCH-IR*). Error bars represent the standard deviation of data. VitC: 0.5 mM vitamin C.

The results showed that knockdown of dUCH decreased the numbers of neurons in the DM (DM-dorsal medial, 2-3 cells), DL1 (DL-dorsal lateral, 1-2 cells), and DL2 (1 cell) clusters (Figures 7(a), 7(d), 7(g)–7(i)) compared to control group. PLE at 2 mg/mL and vitamin C at 0.5 mM did not affect the numbers of DA neurons in control larvae (Figures 7(a)–7(c), 7(g)–7(i)) and were able to ameliorate neurodegeneration in both the DM and DL2 clusters as well as a total of three clusters (Figures 7(d)–7(i)). The results suggested that PLE treatment could decrease the loss of DA neurons in the larval stage.

**Figure 8:**
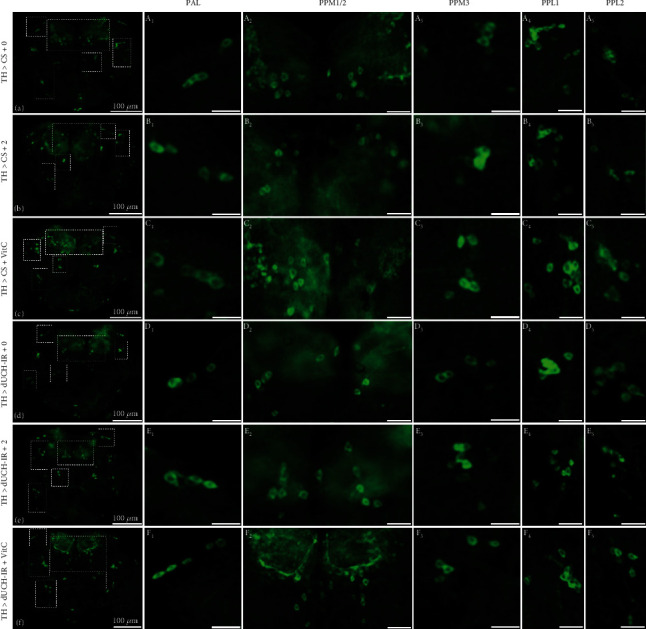
*P. fruticosa* leaf extract reduced degeneration of dopaminergic neurons caused by knockdown of dUCH in 5-day-old flies. (a–f) Representative immunostaining images of whole central brain using antityrosine hydroxylase antibody. The control (TH > CS) and dUCH-knockdown flies (TH > dUCH-IR) were treated with 2 mg/mL of PLE and 0.5 mM vitamin C. (A_1_-F_1_), (A_2_-F_2_), (A_3_-F_3_), (A_4_-F_4_), and (A_5_-F_5_) show dopaminergic neurons in PAL, PPM1/2, PPM3, PPL1, and PPL2 clusters, respectively. Scale bars indicate 50 *μ*m (A_2_-F_2_, A_4_-F_4_, and A_5_-F_5_) and 25 *μ*m (A_1_-F_1_ and A_3_-F_3_). The control line TH > CS (+; +; *TH-GAL4*/+) and the dUCH-knockdown line TH > dUCH-IR (+; +; *TH-GAL4*/*UAS-dUCH-IR*). VitC: 0.5 mM vitamin C.

**Figure 9:**
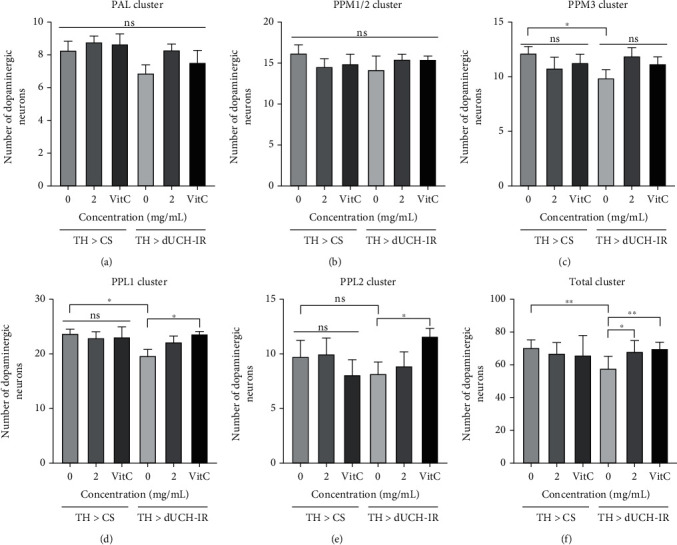
Numbers of dopaminergic neurons in five clusters (PAL, PPM1/2, PPM3, PPL1, and PPL2) and sum of five clusters at 5-day-old flies (a–f). *n* = 8, *t* − test: ^ns^*p* > 0.05, ^∗^*p* < 0.05, and ^∗∗^*p* < 0.01. The control line TH > CS (+; +; *TH-GAL4*/+) and the dUCH-knockdown line TH > dUCH-IR (+; +; *TH-GAL4*/*UAS-dUCH-IR*). Error bars represent the standard deviation of data. VitC: 0.5 mM vitamin C.

Furthermore, the 2 mg/mL of PLE recovered fly climbing ability in the 5-day-old adult stage. Thus, the brains from the 5-day-old flies were collected to examine the effect of PLE on DA neurons. The results illustrated that the numbers of DA neurons in the PPM3 and PPL1 clusters and the total number of DA neurons in five clusters were lower in dUCH-knockdown flies (10, 19, and 57 neurons, resp.) than in control flies (12, 23, and 69 neurons, resp.) (Figures 8(a) (A_3-4_) and 8(d) (D_3-4_); Figures 9(c), 9(d), and 9(f)). Similar to the larval stage, PLE at 2 mg/mL and vitamin C at 0.5 mM did not affect the numbers of DA neurons in the control fly group (Figures 8(a) (A_1-5_), 8(b) (B_1-5_), and 8(c) (C_1-5_); Figure 9) and improved the number of DA neurons in PPL1, PPL2 clusters, and the total of five clusters compared with the untreated flies (Figures 8(d) (D_4-5_), 8(e) (E_4-5_), and 8(f) (C_4-5_); Figures 9(d)–9(f)). The results of this study demonstrated that *P. fruticosa* leaf extract could protect against the degeneration of DA neurons induced by dUCH knockdown in not only the larval stage but also the adult stage, which might result in improvements in locomotive competence of dUCH-knockdown larvae and flies.

## 4. Discussion

Oxidative stress is the cause of many different pathologies. Scientists have demonstrated a link between oxidative stress and Parkinson's disease (PD) [35]. This suggests that antioxidants could be a potential therapy in the treatment of PD. *Polyscias fruticosa* (L.) Harms is not only used as a raw vegetable but also a medicine that promotes health and cures many diseases. Previous studies demonstrated that *P. fruticosa* leaves have many pharmacological effects [4–9]. In this study, we investigated the new pharmacological effect of *P. fruticosa* leaves extract as amelioration of PD symptoms such as locomotor dysfunction and DA damage/loss. The results are well consistent with previous studies in which curcumin and *Portulaca oleracea* L. extract were used as antioxidant sources for treatment on the PD fly model [19, 20]. The results in this study strongly indicated that the *P. fruticosa* leaf extract has a high potential for PD treatment.

Particularly, motor abnormalities in Parkinson's patients are thought to be due to the death of dopaminergic neurons in the SNpc (substantia nigra pars compacta) [36]. In this study, the dUCH knockdown of DA neuron-specific also led to DA neuron degeneration similar to previous studies [19, 20] and other Parkinson's disease models [37–39]. The present results showed that this adverse condition significantly reduced with PLE treatment in both larval and adult stages. Prior reports also showed the potential of medicinal extracts and natural compounds to improve degeneration of DA neurons in some animal models of PD, for example, black sea cucumber (*Holothuria leucospilota*) extracts had the potential to notably improve DA neuron degeneration in 6-OHDA-induced worms [31], Scopoletin could recover dopaminergic neural networks and mobility in *Drosophila* model of PD [40], Ginseng protein prevented mitochondrial dysfunction and neurodegeneration in a fly PINK1 model of PD [41]. Capsaicin [42], *Mucuna pruriens* [43], *Tinospora cordifolia* [44], ursolic acid [45], and chlorogenic acid [46] were also capable of preventing the degeneration of dopamine neurons in the MPTP-induced PD on mouse model by reducing the oxidative stress and neuroinflammation. Korean Red Ginseng also had protective effects against rotenone-induced PD in rat model via several molecular signals [47].

On the other hand, DA neuron degeneration caused long-term unfavorable effects on mobility in both larval and adult stages. Since PLE decreased the degeneration of dopamine-producing neurons, so it improved the crawling capacity of the larvae and climbing capacity of the adult flies. The results obtained were similar to published results using the fly model with the treatment of myricetin, *Centella asiatica* leaf extract, and Majun Baladur (an Unani polyherbal drug) [48–50].

Phytochemical screening gives an idea about the chemical nature of the constituents that have biological effects present in plant extracts [51]. In this study, preliminary analysis by Ciulei's method revealed the presence of volatile oils, free triterpenoids, triterpenoid hydrolysis, alkaloids, flavonoids, anthocyanosides, proanthocyanidins, tannins, saponins, and organic acids in *P. fruticosa* leaves (Table S1). The PD treatment potential of PLE might be due to the presence of polyphenols, flavonoids, and saponins. Dietary intake of plant polyphenols is protective against neurodegeneration was reported previously [52]. Since polyphenols have high antioxidant properties, their consumption might help protect against neurological diseases [52]. The role of polyphenols in improving Parkinson's disease has been demonstrated [53]. Flavonoids are the most studied group of polyphenols with many typical antioxidant properties. In addition, the beneficial effects of flavonoids against Parkinson's disease along with a number of related protective mechanisms have been discovered [54, 55]. Saponins have been investigated in this study as a constituent of *P. fruticosa* leaf extract (Figure 1). Their effects in neurodegenerative, neuropsychiatric, and affective disorders were recently reviewed [56]. In neuroprotection, the role of oleanolic acid and related mechanisms has been shown in many pathological models such as Alzheimer's disease models, Parkinsonian rat models, stem cell differentiation, and brain slice model of neurodegeneration and ischemic stroke [57–60].

However, the excessive consumption of substances with antioxidant properties could cause toxicity and a number of side effects [61]. There is a question of whether *P. fruticosa* leaf extract is toxic and has adverse physiological effects. In the present research, the toxicity of *P. fruticosa* leaf extract was not detected at investigated concentrations in either control flies or dUCH-knockdown flies. Simultaneously, *P. fruticosa* leaf extract also did not adversely affect growth time of flies. This indicates that the PLE's safety is high for further research and therapeutic product development.

## 5. Conclusion

*Polyscias fruticosa* (L.) Harms has been commonly applied in folklore and demonstrated to have many pharmacological effects, especially those in the direction of neuropharmacology. In this study, *P. fruticosa* leaf extract showed its strong potential on PD treatment via ameliorating locomotor ability and reducing dopaminergic neuron degeneration on the transgenic *Drosophila* model of Parkinson's disease. The appropriate concentration of *P. fruticosa* leaf extract at which PD-like phenotypes induced by knockdown of dUCH were improved was 2 mg/mL.

## Figures and Tables

**Table 1:** Phytochemical contents and antioxidant activity of *P. fruticosa* leaf extract.

Extract	PLE
Crude yield (g/100 g RM d. w.)	27.25
TPC (mg GAE/100 mg CE d. w.)	20.57 ± 0.30
TFC (mg QE/g CE d. w.)	8.30 ± 0.27
Oleanolic acid (mg/g d. w.)	23.50 ± 0.20
DPPH inhibition (IC_50_, *μ*g/mL)	77.74 ± 2.73
ABTS inhibition (IC_50_, *μ*g/mL)	37.71 ± 2.25
Reducing power (EC_50_, *μ*g/mL)	141.41 ± 2.25
Lipid peroxidation inhibition (IC_50_, *μ*g/mL)	56.97 ± 0.11

PLE: *P. fruticosa* leaf extract; RM: raw materials; GAE: gallic acid equivalent; QE: quercetin equivalent; CE: crude extract. *n* = 3 and error bars represent the standard deviation of data.

## Data Availability

The datasets generated during the current study are available from the corresponding author on reasonable request.

## Supplementary Materials

Supplementary Materials Detailed information on phytochemical constituents of *P. fruticosa* leaves, antioxidant capacity of *P. fruticosa* leaf extract, the effects of *P. fruticosa* leaf extract on the locomotive ability of control larvae, and the effects of short-term treatment with *P. fruticosa* leaf extract on the climbing ability of 5-day-old adult flies are presented in the supplementary materials listed below: Table S1: phytochemical constituents of *P. fruticosa* leaves. Figure S1: antioxidant capacity of *P. fruticosa* leaf extract. (a, a') DPPH radical scavenging activity of PLE and vitamin C; (b, b') ABTS radical cation decolorization activity of PLE and vitamin C; (c, c') reducing power activity of PLE and vitamin C; (d, d') inhibition of lipid peroxidation of PLE and vitamin C; *n* = 3 and error bars represent the standard deviation of data. Figure S2: the effects of *P. fruticosa* leaf extract on the locomotive ability of control larvae. (a) Average crawling speeds of third-instar larvae. The control line TH-GAL4 (+; +; *TH-GAL4*/+). Error bars represent the standard deviation of data. VitC: 0.5 mM vitamin C. *n* = 45, one-way ANOVA: Tukey's test: ^∗^*p* < 0.05 and ^∗∗^*p* < 0.01. (b) Images show the motion paths of control larvae (TH > CS), control larvae treated with PLE at different concentrations (TH > CS+ 1-16) and 0.5 mM vitamin C (TH > CS + VitC0.5). Figure S3: the effects of short-term treatment with *P. fruticosa* leaf extract on the climbing ability of 5-day-old adult flies. Short-term treatment: the treatment was carried out from the embryonic stage and interrupted after the third larval stage. Population size *N* = 10 and biological replication *n* = 8; Mann-Whitney test, ns: not significant, ^ns^*p* > 0.05 and ^∗∗∗^*p* < 0.001. The control line TH-GAL4 (+; +; *TH-GAL4*/+) and the dUCH-knockdown line TH > dUCH-IR (+; +; *TH-GAL4*/*UAS-dUCH-IR*). Error bars represent the standard deviation of data. VitC: 0.5 mM vitamin C.

## References

[1] Boye A., Osei Owusu A., Koffuor G., Barku V. Y. A., Asiamah E. A., Asante E. (2018). Assessment of Polyscias fruticosa (L.) Harm (Araliaceae) leaf extract on male fertility in male Wistar rats. *Journal of Complementary Medicine Research*.

[2] Vo V. C. (2012). *Dictionary of Vietnamese medicinal plants*.

[3] Nguyen M. P. (2020). Impact of roasting to total phenolic, flavonoid and antioxidant activities in root, bark and leaf of Polyscias fruticosa. *Journal of Pharmaceutical Research International*.

[4] Nguyen T. T. H., Huynh T. M. (2003). In vitro antioxidant effects of Polyscias fruticosa Harms. Araliaceae. *Journal of Medicinal Materials*.

[5] Nguyen T. T. H., Luong K. B. (2001). Research on anti-depressant and anti-stress effects of Polyscias fruticosa. *Journal of Medicinal Materials*.

[6] Nguyen T. T. H., Matsumoto K., Watanabe H. Protective effect of Polyscias fruticosa leaves on social isolation stress-induced brain tissue damage.

[7] Nguyen T. T. H., Nguyen T. A. N. (2004). Study on hepatoprotective effects of Dinh Lang based on the mechanism of antioxidant effect. *Journal of Medicinal Materials*.

[8] Nguyen T. T. H., Tran M. T. Study on effects of Vietnamese medicinal plants on learning and memory.

[9] Nguyen T. T. H., Tran T. M. X. (2008). Memory-improving effect of alcohol extract from Dinh lang leaves (Polyscias fruticosa L. Harms, Araliaceae). *Ho Chi Minh City Journal of Medicine*.

[10] Olanow C. W., Tatton W. G. (1999). Etiology and pathogenesis of Parkinson’s disease. *Annual Review of Neuroscience*.

[11] Dorsey E. R., Constantinescu R., Thompson J. P. (2007). Projected number of people with Parkinson disease in the most populous nations, 2005 through 2030. *Neurology*.

[12] Poewe W., Seppi K., Tanner C. M. (2017). Parkinson disease. *Nature Review Disease Primers*.

[13] Dang T. P. T. (2019). Ubiquitin carboxyl-terminal hydrolase L1 in Parkinson’s. *Ubiquitin Proteasome System - Current Insights into Mechanism Cellular Regulation and Disease*.

[14] Chia S. J., Tan E. K., Chao Y. X. (2020). Historical perspective: models of Parkinson’s disease. *International Journal of Molecular Sciences*.

[15] Stacy M., Bowron A., Guttman M. (2005). Identification of motor and nonmotor wearing-off in Parkinson’s disease: comparison of a patient questionnaire versus a clinician assessment. *Movement Disorders*.

[16] Pandey U. B., Nichols C. D. (2011). Human disease models in Drosophila melanogaster and the role of the fly in therapeutic drug discovery. *Pharmacological Reviews*.

[17] Tran H. H., Dang S. N. A., Nguyen T. T. (2018). Drosophila ubiquitin C-Terminal hydrolase knockdown model of Parkinson’s disease. *Scientific Reports*.

[18] Huynh M. A., Dao M. L., Vuu M. D., Dang T. P. T. (2019). Evaluating dose- and time-dependent effects of vitamin C treatment on a Parkinson’s disease fly model. *Parkinson's Disease*.

[19] Nguyen T. T., Vuu M. D., Huynh M. A., Yamaguchi M., Tran L. T., Dang T. P. T. (2018). Curcumin effectively rescued Parkinson’s disease-like phenotypes in a novel Drosophila melanogaster model with dUCH knockdown. *Oxidative Medicine and Cellular Longevity*.

[20] Truong H. K. T., Huynh M. A., Vuu M. D., Dang T. P. T. (2019). Evaluating the potential of Portulaca oleracea L. for Parkinson’s disease treatment using a Drosophila model with dUCH-knockdown. *Parkinson's Disease*.

[21] Lefahal M., Zaabat N., Ayad R. (2018). In vitro assessment of total phenolic and flavonoid contents, antioxidant and photoprotective activities of crude methanolic extract of aerial parts of Capnophyllum peregrinum (L.) Lange (Apiaceae) growing in Algeria. *Medicines (Basel)*.

[22] Tran V. T., Tran T. H. H., Nguyen T. D. (2016). Validated high performance liquid chromatography method for quantification of a major saponin in Polyscias fruticosa. *Journal of Multidisciplinary Engineering Science and Technology*.

[23] Oyaizu M. (1986). Studies on product of browning reaction prepared from glucose amine. *Japan Journal of Nutrition*.

[24] Kızıl G., Kızıl M., Yavuz M., Emen S., Hakimoğlu F. (2008). Antioxidant activities of ethanol extracts of Hypericum triquetrifolium and Hypericum scabroides. *Pharmaceutical Biology*.

[25] Nichols C. D., Becnel J., Pandey U. B. (2012). Methods to assay Drosophila behavior. *Journal of Visualized Experiments*.

[26] Ali Y. O., Escala W., Ruan K., Zhai R. G. (2011). Assaying locomotor, learning, and memory deficits in Drosophila models of neurodegeneration. *Journal of Visualized Experiments*.

[27] Dang T. P. T., Phan N. T. A., Yamaguchi M., Tran L. T. (2012). Overexpression of ubiquitin carboxyl terminal hydrolase impairs multiple pathways during eye development in Drosophila melanogaster. *Cell and Tissue Research*.

[28] Panche A. N., Diwan A. D., Chandra S. R. (2016). Flavonoids: an overview. *Journal of Nutritional Science*.

[29] Kumar G. P., Khanum F. (2012). Neuroprotective potential of phytochemicals. *Pharmacognosy Reviews*.

[30] Venkatesan R., Ji E., Kim S. Y. (2015). Phytochemicals that regulate neurodegenerative disease by targeting neurotrophins: a comprehensive review. *Biomed Research International*.

[31] Malaiwong N., Chalorak P., Jattujan P. (2019). Anti-Parkinson activity of bioactive substances extracted from Holothuria leucospilota. *Biomedicune & Pharmacotherapy*.

[32] Qian Y. H., Liu Y., Hu H. T., Ren H. M., Chen X. L., Xu J. H. (2002). The effects of the total saponin of Dipsacus asperoides on the damage of cultured neurons induced by beta-amyloid protein 25-35. *Anatomical Science International*.

[33] Rastogi M., Ojha R. P., Prabu P. C., Devi B. P., Agrawal A., Dubey G. P. (2012). Prevention of age-associated neurodegeneration and promotion of healthy brain ageing in female Wistar rats by long term use of bacosides,. *Biogerontology*.

[34] Feany M. B., Bender W. W. (2000). A Drosophila model of Parkinson’s disease. *Nature*.

[35] Chen X., Guo C., Kong J. (2012). Oxidative stress in neurodegenerative diseases. *Neural Regeneration Research*.

[36] Surmeier D. J. (2018). Determinants of dopaminergic neuron loss in Parkinson’s disease. *The FEBS Journal*.

[37] Faust K., Gehrke S., Yang Y., Yang L., Beal M. F., Lu B. (2009). Neuroprotective effects of compounds with antioxidant and anti-inflammatory properties in a Drosophila model of Parkinson’s disease. *BMC Neuroscience*.

[38] Jeong J. S., Piao Y., Kang S. (2018). Triple herbal extract DA-9805 exerts a neuroprotective effect via amelioration of mitochondrial damage in experimental models of Parkinson’s disease. *Scientific Reports*.

[39] Liu Z., Li T., Yang D., Smith W. W. (2013). Curcumin protects against rotenone-induced neurotoxicity in cell and Drosophila models of Parkinson’s disease. *Advances in Parkinson'S Disease*.

[40] Pradhan P., Majhi O., Biswas A., Joshi V. K., Sinha D. (2020). Enhanced accumulation of reduced glutathione by Scopoletin improves survivability of dopaminergic neurons in Parkinson’s model. *Cell Death & Disease*.

[41] Liu M., Yu S., Wang J. (2020). Ginseng protein protects against mitochondrial dysfunction and neurodegeneration by inducing mitochondrial unfolded protein response in Drosophila melanogaster PINK1 model of Parkinson’s disease. *Journal of Ethnopharmacology*.

[42] Chung Y. C., Baek J. Y., Kim S. R. (2017). Capsaicin prevents degeneration of dopamine neurons by inhibiting glial activation and oxidative stress in the MPTP model of Parkinson’s disease. *Experimental & Molecular Medicine*.

[43] Rai S. N., Birla H., Singh S. S. (2017). Mucuna pruriens protects against MPTP intoxicated neuroinflammation in Parkinson’s disease through NF-*κ*B/pAKT signaling pathways. *Frontiers in Aging Neuroscience*.

[44] Birla H., Rai S. N., Singh S. S. (2019). Tinospora cordifolia suppresses neuroinflammation in Parkinsonian mouse model. *Neuromolecular Medicine*.

[45] Rai S. N., Zahra W., Singh S. S. (2019). Anti-inflammatory activity of ursolic acid in MPTP-induced Parkinsonian mouse model. *Neurotoxicity Research*.

[46] Singh S. S., Rai S. N., Birla H. (2018). Effect of chlorogenic acid supplementation in MPTP-intoxicated mouse. *Frontiers in Pharmacology*.

[47] Zaafan M. A., Abdelhamid A. M., Ibrahim S. M. (2019). The protective effect of Korean red ginseng against rotenone-induced Parkinson’s disease in rat model: modulation of nuclear factor-*κβ* and Caspase-3. *Current Pharmaceutical Biotechnology*.

[48] Siddique Y. H., Naz F., Jyoti S. (2014). Effect of Centella asiatica leaf extract on the dietary supplementation in transgenic Drosophila model of Parkinson’s disease. *Parkinson's Disease*.

[49] Siddique Y. H., Naz F., Rashid M. (2019). Effect of Majun Baladur on life span, climbing ability, oxidative stress and dopaminergic neurons in the transgenic Drosophila model of Parkinson's disease. *Heliyon*.

[50] Ara G., Afzal M., Jyoti S., Siddique Y. H. (2017). Effect of myricetin on the transgenic Drosophila model of Parkinson’s disease. *Bulletin of Faculty of Pharmacy, Cairo University*.

[51] Mendoza N., Silva E. M. E. (2018). Introduction to phytochemicals: secondary metabolites from plants with active principles for pharmacological importance, phytochemicals-source of antioxidants and role in disease prevention. *Phytochemicals: Source of Antioxidants and Role in Disease Prevention*.

[52] Pandey K. B., Rizvi S. I. (2009). Plant polyphenols as dietary antioxidants in human health and disease. *Oxidative Medicine and Cellular Longevity*.

[53] Kujawska M., Jodynis-Liebert J. (2018). Polyphenols in Parkinson’s disease: a systematic review of in vivo studies. *Nutrients*.

[54] Jung U. J., Kim S. R. (2018). Beneficial effects of flavonoids against Parkinson’s disease. *Journal of Medicinal Food*.

[55] Magalingam K. B., Radhakrishnan A. K., Haleagrahara N. (2015). Protective mechanisms of flavonoids in Parkinson’s disease. *Oxidative Medicine and Cellular Longevity*.

[56] Sun A., Xu X., Lin J., Cui X., Xu R. (2015). Neuroprotection by saponins. *Phytotherapy Research*.

[57] Ndlovu B. C., Daniels W. M., Mabandla M. V. (2014). Oleanolic acid enhances the beneficial effects of preconditioning on PC12 cells. *Parkinson's Disease*.

[58] Sen A. (2020). Prophylactic and therapeutic roles of oleanolic acid and its derivatives in several diseases. *World Journal of Clinical Cases*.

[59] Wang X., Ye X. L., Liu R. (2010). Antioxidant activities of oleanolic acid in vitro: possible role of Nrf 2 and MAP kinases. *Chemico-Biological Interactions*.

[60] Gudoityte E., Arandarcikaite O., Mazeikiene I., Bendokas V., Liobikas J. (2021). Ursolic and oleanolic acids: plant metabolites with neuroprotective potential. *International Journal of Molecular Sciences*.

[61] Salehi B., Martorell M., Arbiser J. L. (2018). Antioxidants: positive or negative actors?. *Biomolecules*.

