# Supplementary material for: Therapeutic Potential of Polyscias fruticosa (L.) Harms Leaf Extract for Parkinson's Disease Treatment by Drosophila melanogaster Model
Source: Oxid Med Cell Longev. 2022 May 19;2022:5262677. doi: 10.1155/2022/5262677 (PMC9135533; doi:10.1155/2022/5262677)
Supplement: Supplementary Materials — Detailed information on phytochemical constituents of P. fruticosa leaves, antioxidant capacity of P. fruticosa leaf extract, the effects of P. fruticosa leaf extract on the locomotive ability of control larvae, and the effects of short-term treatment with P. fruticosa leaf extract on the climbing ability of 5-day-old adult flies are presented in the supplementary materials listed below: Table S1: phytochemical constituents of P. fruticosa leaves. Figure S1: antioxidant capacity of P. fruticosa leaf extract. (a, a') DPPH radical scavenging activity of PLE and vitamin C; (b, b') ABTS radical cation decolorization activity of PLE and vitamin C; (c, c') reducing power activity of PLE and vitamin C; (d, d') inhibition of lipid peroxidation of PLE and vitamin C; n = 3 and error bars represent the standard deviation of data. Figure S2: the effects of P. fruticosa leaf extract on the locomotive ability of control larvae. (a) Average crawling speeds of third-instar larvae. The control line TH-GAL4 (+; +; TH-GAL4/+). Error bars represent the standard deviation of data. VitC: 0.5 mM vitamin C. n = 45, one-way ANOVA: Tukey's test: ∗p < 0.05 and ∗∗p < 0.01. (b) Images show the motion paths of control larvae (TH > CS), control larvae treated with PLE at different concentrations (TH > CS+ 1-16) and 0.5 mM vitamin C (TH > CS + VitC0.5). Figure S3: the effects of short-term treatment with P. fruticosa leaf extract on the climbing ability of 5-day-old adult flies. Short-term treatment: the treatment was carried out from the embryonic stage and interrupted after the third larval stage. Population size N = 10 and biological replication n = 8; Mann-Whitney test, ns: not significant, nsp > 0.05 and ∗∗∗p < 0.001. The control line TH-GAL4 (+; +; TH-GAL4/+) and the dUCH-knockdown line TH > dUCH-IR (+; +; TH-GAL4/UAS-dUCH-IR). Error bars represent the standard deviation of data. VitC: 0.5 mM vitamin C. [file 5262677.f1.pdf]

Supplementary Materials

Table S1: Phytochemical constituents of *P. fruticosa* leaves

Metabolites	Diethyl ether extract	Ethanol extract		Aqueous extract		Presence
		Non-hydrolysis	Hydro-lysis	Non-hydrolysis	Hydro-lysis	
Lipids	-	ND	ND	ND	ND	-
Carotenoids	-	ND	ND	ND	ND	-
Volatile oils	+	ND	ND	ND	ND	+
Free triterpenoids	+	ND	ND	ND	+	+
Triterpenoid hydrolysis	ND	ND	+	ND	+	+
Alkaloids	-	+	ND	+	ND	+
Coumarins	-	-	ND	-	ND	-
Anthraglycosides		ND	ND	-	ND	-
Flavonoids	-	+	+	+	+	+
Anthocyanosides	ND	+	ND	+	ND	+
Proanthocyanidins	ND	+	ND	+	ND	+
Tannins	ND	+	ND	+	ND	+
Saponins	ND	+	ND	+	ND	+
Organic acids	ND	+	ND	+	ND	+
Reducing agents	ND	+	ND	+	ND	+
Polyuronics	ND		ND	-	ND	-

(+): the presence; (−): the absence; ND: not determined

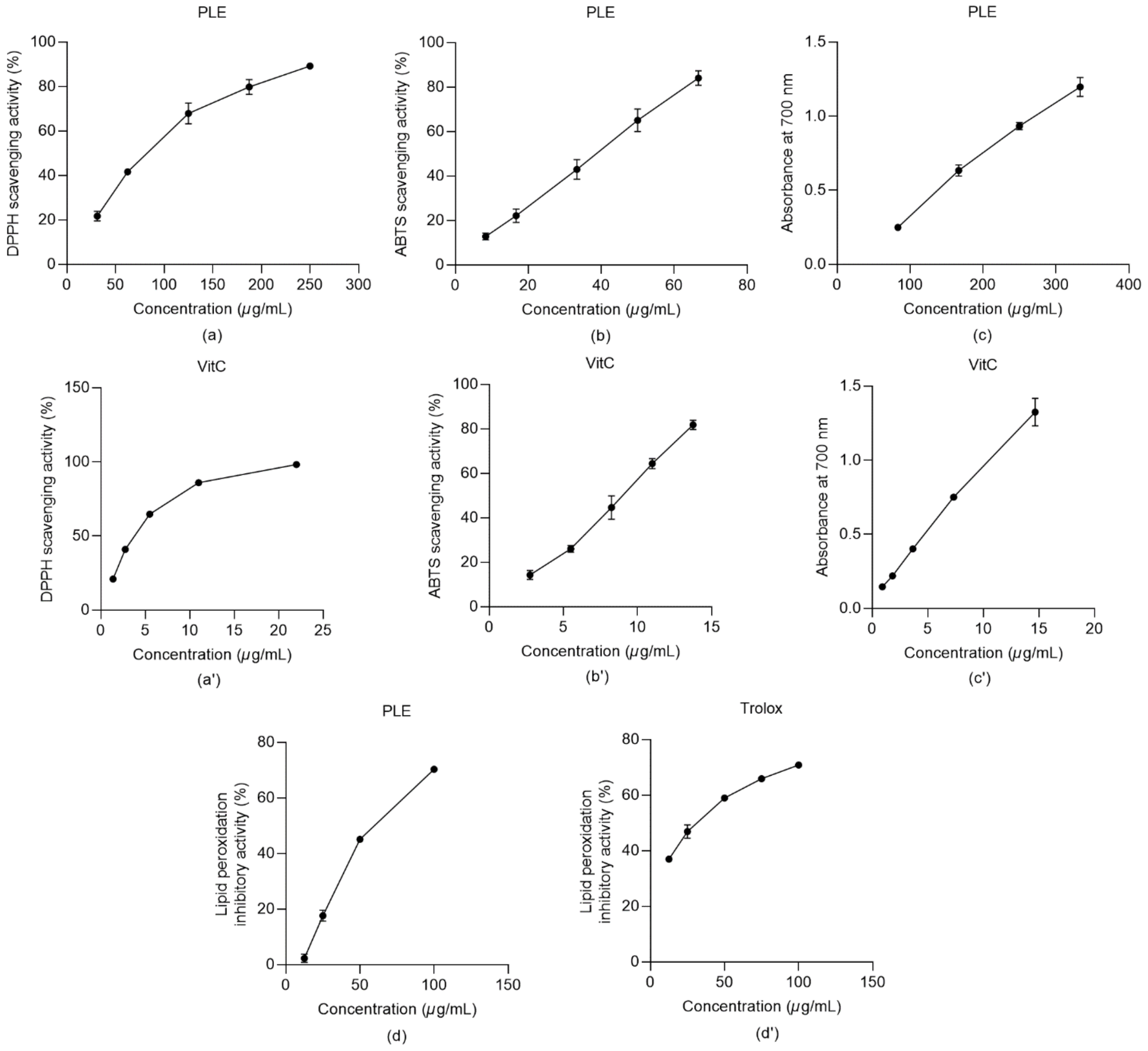

Figure S1: Antioxidant capacity of *P. fruticosa* leaf extract. (a, a’) DPPH radical scavenging activity of PLE and vitamin C; (b, b’) ABTS radical cation decolorization activity of PLE and vitamin C; (c, c’) Reducing power activity of PLE and vitamin C; (d, d’) Inhibition of lipid peroxidation of PLE and vitamin C; $n = 3$ and error bars represent the standard deviation of data.

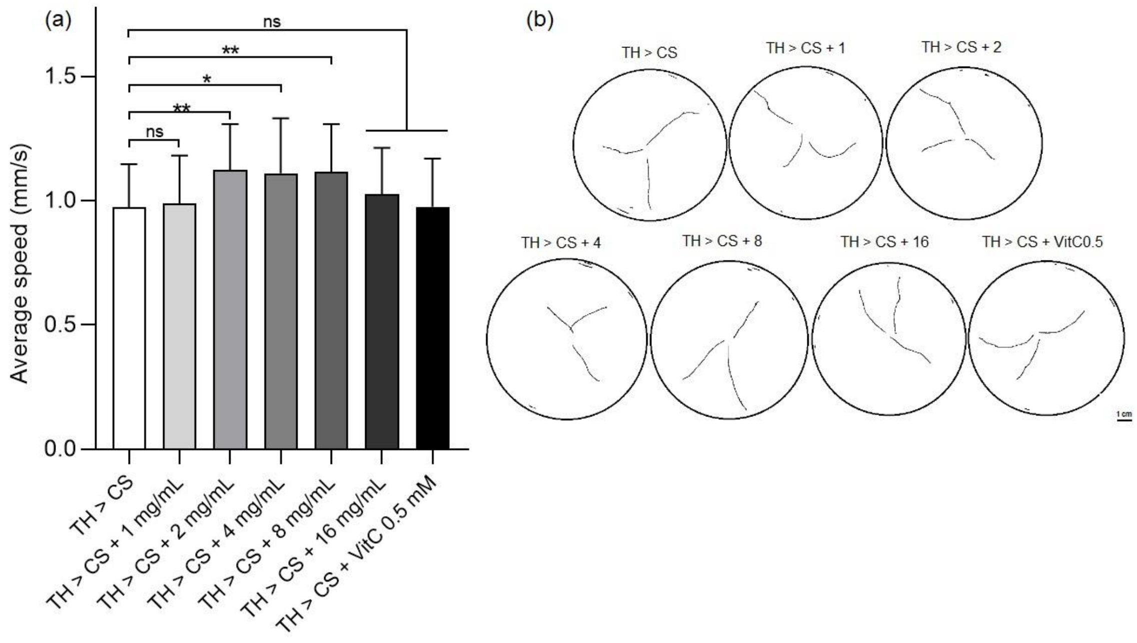

Figure S2: The effects of *P. fruticosa* leaf extract on the locomotive ability of control larvae. (a) Average crawling speeds of third-instar larvae. The control line TH-GAL4 (+; +; *TH-GAL4*/+). Error bars represent the standard deviation of data. VitC: 0.5 mM vitamin C. $n = 45$, one-way ANOVA: Tukey's test: $^{*}p < 0.05$, $^{**}p < 0.01$. (b) Images show the motion paths of control larvae (TH > CS), control larvae treated with PLE at different concentrations (TH > CS + 1-16) and 0.5 mM vitamin C (TH > CS + VitC0.5).

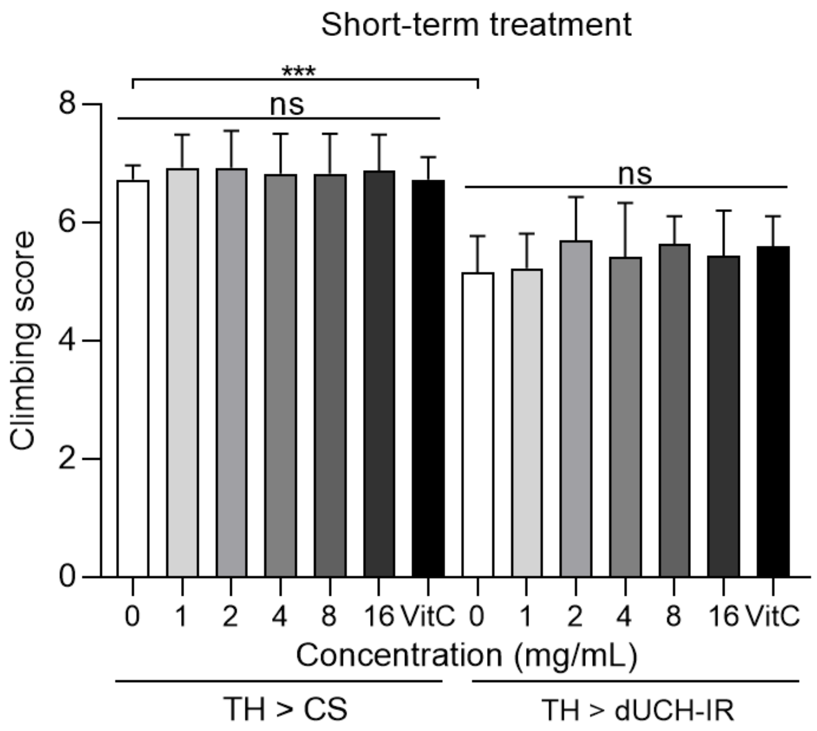

Figure S3: The effects of short-term treatment with *P. fruticosa* leaf extract on the climbing ability of 5-day-old adult flies. Short-term treatment: the treatment was carried out from the embryonic stage and interrupted after the third larval stage. Population size N = 10 and biological replication $n = 8$; Mann-Whitney test, ns: not significant, $^{ns}p > 0.05$, $^{***}p < 0.001$. The control line TH-GAL4 (+; +; *TH-GAL4*/+) and the dUCH-knockdown line TH > dUCH-IR (+; +; *TH-GAL4*/*UAS-dUCH-IR*). Error bars represent the standard deviation of data. VitC: 0.5 mM vitamin C.
